# Significant healthcare burden and life cost of spinal muscular atrophy: real-world data

**DOI:** 10.1007/s10198-022-01548-5

**Published:** 2022-11-20

**Authors:** Sophelia H. S. Chan, Carlos K. H. Wong, Tingting Wu, Wilfred Wong, Michael K. L. Yu, Ivan C. H. Au, Godfrey C. F. Chan

**Affiliations:** 1 Department of Paediatrics and Adolescent Medicine, Li Ka Shing Faculty of Medicine, The University of Hong Kong, Hong Kong Special Administrative Region, China; 2 Department of Pharmacology and Pharmacy, Li Ka Shing Faculty of Medicine, The University of Hong Kong, Hong Kong Special Administrative Region, China; 3 Department of Family Medicine and Primary Care, LKS Faculty of Medicine, The University of Hong Kong, Hong Kong SAR, China

**Keywords:** Spinal muscular atrophy, Healthcare burden, Mortality, Newborn screening, Disease modifying treatment

## Abstract

**Objectives:**

The aim of this study is to quantify the mortality rate, direct healthcare costs, and cumulative life costs of pediatric patients with spinal muscular atrophy (SMA) type 1, type 2, and type 3 born in Hong Kong.

**Methods:**

Data were collected from genetically confirmed SMA patients born in or after 2000 from the Hospital Authority medical database. Patients were followed up from birth until they died, left Hong Kong, reached 18 years, or initiated disease-modifying treatment. Study outcomes included incidence risks of mortality, cumulative direct medical costs—attendances of special outpatient clinics, emergency department, allied health services, and mean length of stay in hospitals over time. Total direct medical costs were calculated as unit costs multiplied by utilization frequencies of corresponding healthcare services at each age.

**Results:**

Seventy-one patients with SMA were included. Over a median follow-up period of 6 years, the overall incidence rate of death was 5.422/100 person-years (95%CI 3.542–7.945/100 person-years). 67.7% and 11% of deaths occurred in SMA1 and SMA2 groups, respectively. The median age of death was 0.8 years in SMA1 and 10.9 years in SMA2. The mean cumulative direct medical costs in overall SMA, SMA1, SMA2 and SMA3 groups per patient were US$935,570, US$2,393,250, US$413,165, and US$40,735, respectively.

Interpretation: Our results confirmed a significantly raised mortality and extremely high healthcare burden for patients with SMA especially SMA type 1 and 2 without disease-modifying treatment. Study evaluating health and economic impact of newborn screening and early treatment is needed.

**Supplementary Information:**

The online version contains supplementary material available at 10.1007/s10198-022-01548-5.

## Background

Spinal muscular atrophy (SMA) is a group of hereditary neuromuscular diseases distinguished by the degeneration of motor neurons and/or axons in the spinal cord and brainstem, leading to progressive muscle weakness and wasting and causing significant disability [1]. The most common cause of SMA is a mutation in the survival motor neuron 1 (*SMN1*) gene on chromosome 5, with most patients (95%) having a homozygous exon 7 deletion [2]. As one of the most common and severe neuromuscular diseases in pediatric patients, SMA has an incidence of approximately 1 in 11,000 live births [3] and an average carrier frequency of 1 in 50 [3–6].

SMA is a clinically heterogenous disease with a spectrum of clinical presentations. In most patients, symptoms begin during infancy or early childhood. There are three main clinical subtypes of SMA, which are differentiated by the age at onset of symptoms and the best motor milestones achieved [7]. The severe SMA type 1 (SMA1), which accounts for about 60% of total SMA cases, is the leading cause of infant mortality with most of them died before the age of 2 years without the initiation of respiratory support [7]. Affected babies are generally normal at birth and then develop symptoms, such as muscle weakness, weak cry, breathing difficulties and feeding problems within the first 6 months of life [7]. They never achieve independent sitting, and their generalized weakness progresses rapidly [8]. In natural history cohorts, the median age of death was 8 months, and the median age of reaching death or requiring ≥ 16 h per day of ventilation was 13.5 months [9, 10]. Children with SMA type 2 (SMA2), which accounts for 25–30% of total SMA cases, have an intermediate severity and can sit alone but cannot stand or walk unaided. Some of them eventually lose their ability to sit and develop feeding difficulties and severe respiratory problems. Musculoskeletal complications including hip subluxation, hip dislocation, and kyphoscoliosis are common in children with SMA type 1 and type 2. Children with SMA type 3 (SMA3) have a milder presentation and can walk unaided. However, around 50% will eventually lose their ambulation, usually during adolescence [11].

In the past, there were no effective treatments for SMA, and the standard of care required, but was not limited to, the specialties of neurology, respiratory, orthopedic, gastrointestinal, nutritional, physical therapy, occupational therapy, and speech therapy [12–15]. Following US Food and Drug Administration (FDA) approval of three novel therapies—the antisense oligonucleotide nusinersen (approved in December 2016, Biogens Inc, Cambridge MA), the gene replacement therapy onasemnogene abeparvovec-xioi (approved in May 2019, Novartis), and the small molecules risdiplam (approved in August 2020, Roche/Genentech), the SMA treatment paradigm shifted. These novel therapies have been shown to significantly improve the clinical outcomes of young patients with SMA when the treatment is started at early age [16–19]. However, these treatments are also highly expensive. Real-world data on the economic burden and healthcare costs of SMA prior to the availability of these disease-modifying treatments (DMTs) are, therefore, very important when considering the impact of no DMTs on the medical care costs. Currently, long-term mortality and economic data of SMA are still limited, and modeling analyses are frequently used for estimations.

The aim of this study is to quantify the mortality rate, direct healthcare costs, and cumulative life costs for all paediatric patients with SMA1, SMA2, SMA3 born in or after 2000 in Hong Kong. The healthcare cost calculation is based on actual healthcare service utilization by each patient from birth until either the age of 18 years, death, emigration, or the initiation of DMTs. This study uses the real-world data of the SMA patients to provide mortality data and data on the economic burdens of our pediatric patients with SMA, thus bridging current research gaps.

## Methods

### Study population

In this retrospective cohort study, patients were recruited from the Hong Kong Hospital Authority (HA) database. HA is a statutory body that provides public healthcare services, and data from HA has been widely incorporated in high-quality research. Eligible patients were born in the year 2000 and later up till 2019, and had a diagnosis of SMA with genetic confirmation. Patients were followed-up from birth and later censored if they died, left Hong Kong, reached 18 years of age, or initiated disease-modifying treatment (e.g., nusinersen), whichever occurred first. Data were extracted on 22^nd^ March 2021.

### Outcomes measures

The primary outcomes were the incidence risks of mortality; cumulative direct medical costs of patients with SMA from birth to 18 years; the number of attendances of special outpatient clinics (SOPC), accident and emergency (A&E) department, and allied health services; and mean length of stay in hospitals, including general wards, intensive care units [ICU], and high-dependency units [HDU]) over time. All the outcomes listed were measured for all patients and by SMA types.

### Estimation of cumulative direct medical costs

Costs were calculated with a micro-costing method from a public healthcare provider’s perspective. We referred to the 2017 Hong Kong Government Gazette and Hospital Authority Ordinance (public charges to non-eligible patients and private charges) for unit costs of healthcare services [20] (Supplementary Table 1). Total direct medical costs were calculated for each patient as unit costs multiplied by utilization frequencies of the corresponding healthcare services at each age. The cumulative costs were measured from the date of birth to up to the age of 18 or other censoring causes described earlier and presented in US Dollar (USD) converted from Hong Kong Dollars (HKD) pegged at 1 USD equal or close to 7.8 HKD.

We declare that the study was conducted in accordance with the ethical standards set by the University of Hong Kong (HKU)/HK West Cluster Institutional Review Board (UW21-531).

### Statistical analysis

Demographic information, including the year of birth, sex, death, and initiation of nusinersen treatment, was displayed in numbers and percentages. In addition, the utilization frequency of each healthcare service and length of stay was counted by age and virtualized in line charts and tables. The healthcare resource utilization and length of stay in a hospital ward were counted as it occurred and were not annualized for patients whose follow-up duration (in years) was not an integer. Incidence rates (IRs) of all-cause mortality were assessed in all patients and further estimated by SMA types. Cox’s proportional regression models were also used to calculate hazard ratios (HRs) for all-cause mortality. All statistical analyses were performed by STATA version 16.0 (StataCorp LP, College Station, Texas). The Kaplan–Meier method was used to calculate survival probability and plot the survival curve for patients with SMA type 1 born at or after January 2000 till December 2019. The analysis was using the primary outcome—age at death, and the data were censored if the patients left Hong Kong, initiated diseased-modifying treatment, or reached the age of 18 years. SPSS version 26 was used. All significance tests were two‐tailed, and *P* values < 0.05 were taken to indicate statistical significance.

## Results

### Patient characteristics

This study included 71 pediatric patients with SMA (34 SMA1, 27 SMA2, and 10 SMA3). Most included patients (71.8%) were born between 2006 and 2015 with slightly more female patients (54.9%) than male. Around one-third of patients died during the observation period. Twenty-two patients (31.0%) initiated nusinersen therapy (Table 1).

**Table 1:** Characteristics of study population

Characteristics	Overall (N = 71) N (%)	SMA Type 1 (N = 34) N (%)	SMA Type 2 (N = 27) N (%)	SMA Type 3 (N = 10) N (%)
Year of birth				
Before 2006	10 (14.1%)	2 (5.9%)	5 (18.5%)	3 (30.0%)
2006–2010	25 (35.2%)	16 (47.1%)	7 (25.9%)	2 (20.0%)
2011–2015	26 (36.6%)	11 (32.4%)	11 (40.7%)	4 (40.0%)
2016–2019	10 (14.1%)	5 (14.7%)	4 (14.8%)	1 (10.0%)
Sex				
Male	32 (45.1%)	14 (41.2%)	12 (44.4%)	6 (60.0%)
Female	39 (54.9%)	20 (58.8%)	15 (55.6%)	4 (40.0%)
Death	26 (36.6%)	23 (67.7%)	3 (11%)	0 (0.0%)
Nusinersen	22 (31.0%)	8 (23.5%)	9 (33.3%)	5 (50.0%)

*SMA* Spinal Muscular Atrophy

Risks of mortality.

**Fig. 1:**
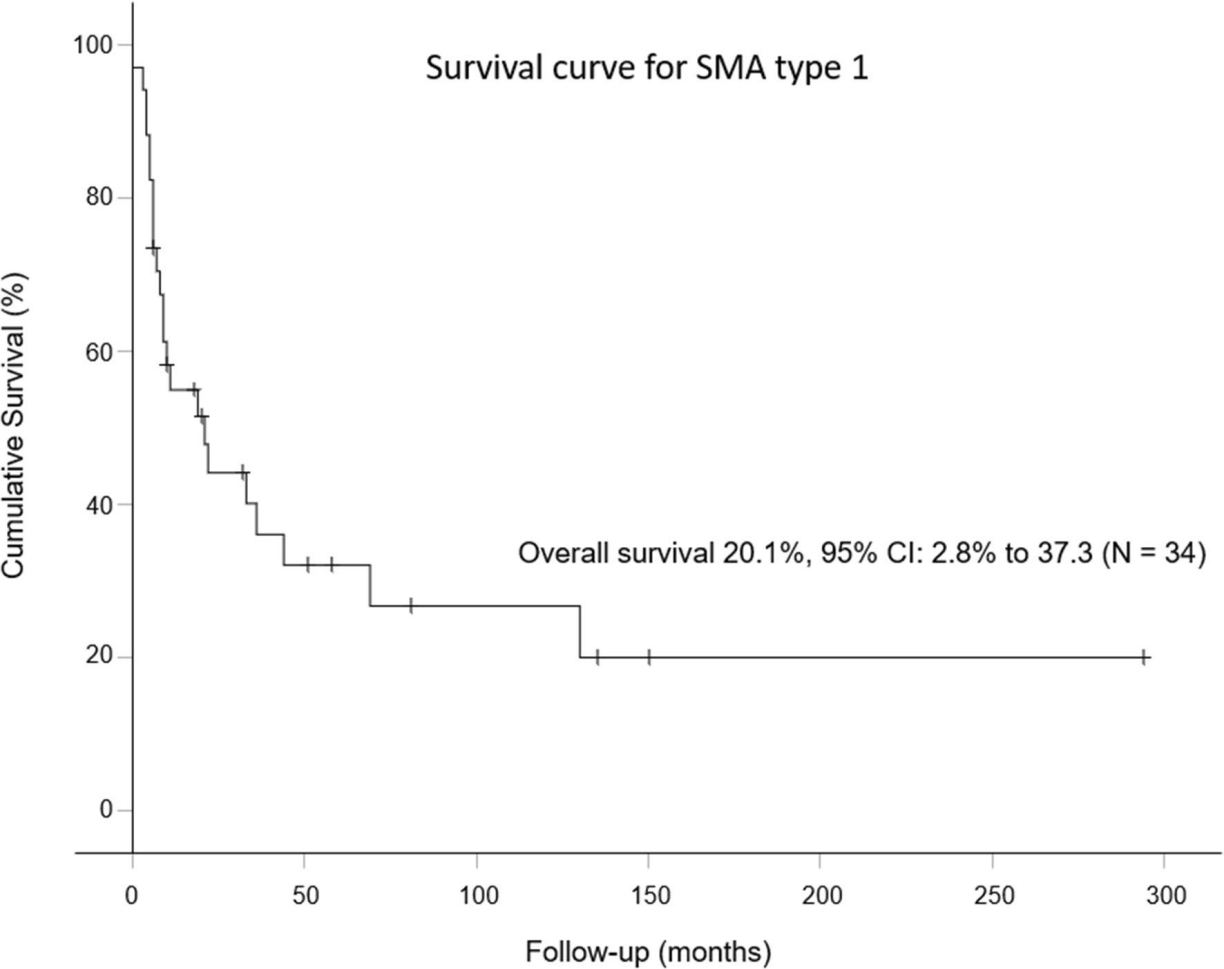
Kaplan–Meier survival plot of spinal muscular atrophy type 1 patients

Over a median follow-up period of 6 years with 480 person-years (Table 2), 23 (36.6%) patients died during the observation period overall. The overall incidence rate of death was 5.422/100 person-years (95%CI 3.542–7.945/100 person-years). Twenty-three (67.7%) and three (11%) deaths occurred in SMA1 and SMA2 groups, respectively, with incidence rates of 20.335/100 person-years (95%CI 12.891–30.512/100 person-years) and 1.161/100 person-years (95%CI 0.239–3.393/100 person-years), respectively. The median age of death was 0.8 years in SMA1 and 10.9 years in SMA2. There were no recorded deaths in the SMA3 group. Patients living with SMA2 had significantly lower risks of all-cause mortality than patients with SMA1 (HR = 0.078, 95%CI 0.026–0.235, *P* < 0.001) (Table 2). The survival plot for SMA type 1 using death as the event and censored if the patients left Hong Kong, initiated diseased-modifying treatment, or reached the age of 18 years is illustrated in Fig. 1. The overall survival was 20.1% (95%CI 2.8–37.3%; number of patients = 34).

**Table 2:** Incidence risks of all-cause mortality

SMA Type	Cumulative incidence		Crude incidence rate (Cases/100 person-years)			Median follow-up periods (years)	Mean follow-up periods (years)	Median time to event (years)	Mean time to event (years)	Hazard ratio	95%CI	*P* value
	Cases with event	Rate	Estimate	95%CI*	Person-years							
Overall	26	0.366	5.422	(3.542, 7.945)	480	5.7	6.8	0.8	2.8			
1	23	0.676	20.335	(12.891, 30.512)	113	1.7	3.3	0.8	1.7	(reference)		
2	3	0.111	1.161	(0.239, 3.393)	258	9.4	9.6	10.9	11.4	0.078	(0.026, 0.235)	< 0.001
3	0	0.000	0.000	NA	108	10.5	10.8	NA	NA	NA	NA	NA

*SMA* Spinal Muscular Atrophy, *CI* confidence interval, *NA* not applicable

### Cumulative direct medical costs and costs of healthcare services

**Fig. 2:**
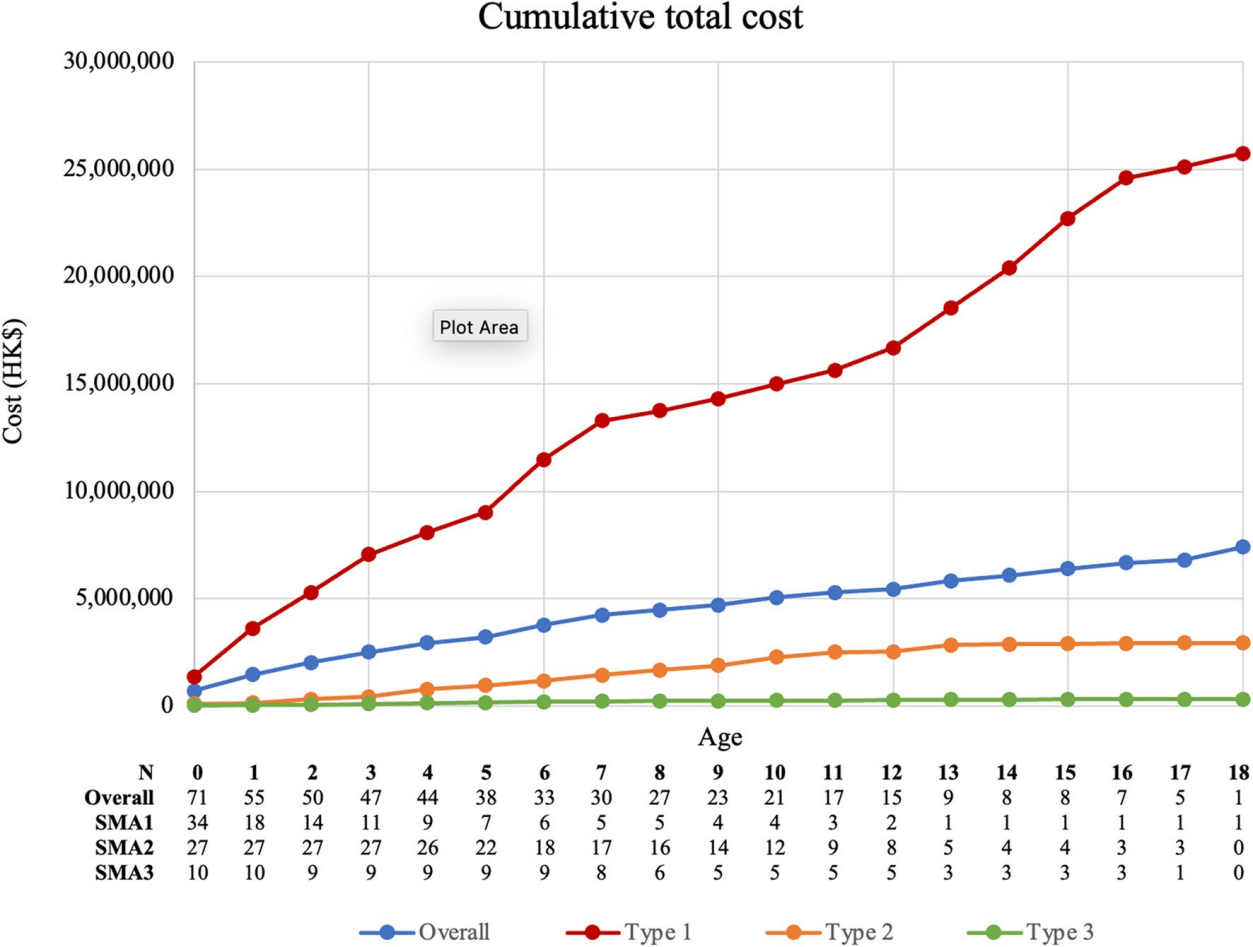
Cumulative total direct medical costs of patients with SMA by age. Cumulative total direct medical costs (HK$) of patients with SMA by age in the four groups (overall SMA group, SMA type 1 group, SMA type 2 group and SMA type 3 group)

Following the micro-costing approach, the mean cumulative direct medical costs per patient were HK$7,297,440 (US $935,570) in overall SMA group: HK$18,667,345 (US $2,393,250) in SMA1, HK$3,222,682 (US $413,165) in SMA2, and HK$317,727 (US $40,735) in SMA3 from birth to 18 years (Fig. 1). The largest component of the direct medical costs was hospitalization (HK$6,931,934) (US $888,709) (95.0%), followed by SOPC (HK$285,218) (US $36,566) (3.91%), operations or procedures (HK$163,485) (US $20,959) (2.24%), and A&E services (HK$26,106) (US $3,346) (0.36%) (Fig. 2; Supplementary Table 2). The cumulative total costs of patients with SMA1 increased more rapidly with age than those of SMA2 and SMA3 groups (Fig. 2).

Overall, higher annual direct medical costs were incurred in the first 3 years after birth, ranging from HK$559,503 (USD $71,731) to HK$759,091 (USD $97,320). After this, annual direct costs gradually decreased with some fluctuations as patients grew older, dropping to HK$119,361 (US $15,303) when patients are 17 years. Hospitalizations accounted for the greatest proportion of direct medical costs each year (Supplementary Table 2).

### Frequency of utilization of healthcare services

**Fig. 3:**
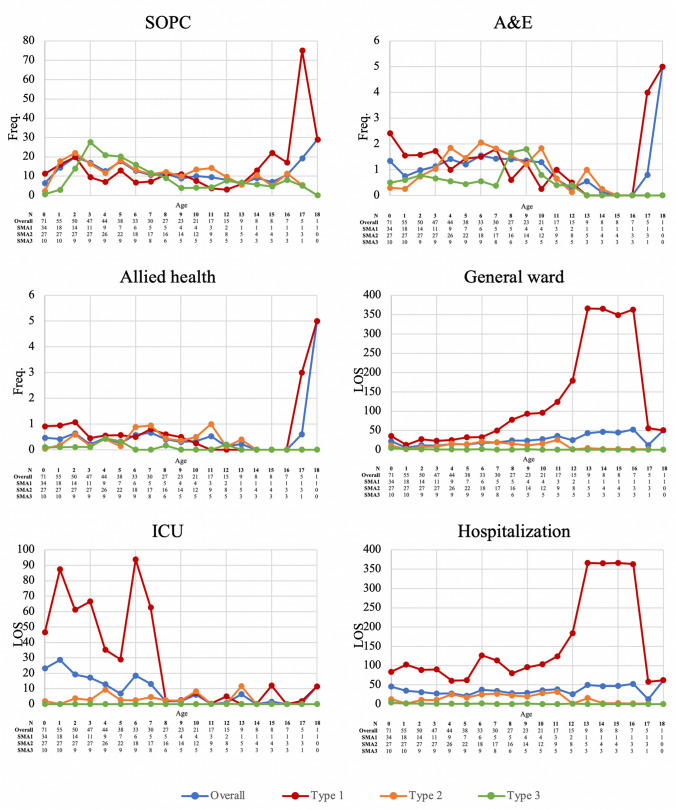
Utilization frequency of healthcare services of patients with SMA by age. Utilization frequency of healthcare services (including SOPC, A&E, allied health, general ward, ICU and hospitalization) of patients with SMA by age in the four groups (overall SMA group, SMA type 1 group, SMA type 2 group and SMA type 3 group)

Figure 3 and Supplementary Table 3 present the frequency of healthcare utilization and length of stay in hospital wards at each age. Patients with SMA1 visited SOPC more than 10 times between the ages of 0 to 2 and 15 to 18. In patients with SMA2, frequent SOPC visits (8–23 visits per year) occurred in and after 1 year, while patients with SMA3 visited SOPC most intensively (12–28 visits per year) between 2 and 7 years. In all SMA groups, attendances to A&E departments (0–2 admissions per year) and allied health professional services (≤ 1 time) were rare over the years, except for one patient with SMA1 who suddenly increased utilization at ages 17 and 18. As for hospitalization, patients with SMA1 spent 39–97 days annually in hospital wards between birth and 7 years, over half of which were in ICUs. Moreover, while admissions to general wards in the SMA1 group increased dramatically from 56 days at 8 years to 363 days at 16 years, the length of stay in an ICU decreased to less than 6 days after age 8. The mean length of stay in general wards for patients with SMA3 was 4 days in their year of birth, and they spent 0–2 days annually in the subsequent years. No patients with SMA3 were admitted to ICUs in our study period. The mean length of stay for patients with SMA2 was longer than the SMA3 group but shorter than the SMA1 group (Fig. 3).

## Discussion

This study measured the mortality and economic burden of patients with SMA using real-world data with follow-up from birth up to 18 years. The mortality rate in the SMA1 was 67.7%, with an incidence rate of 20.355 per 100 person-years, followed by 11% in the SMA2 group with an incidence rate of 1.161 per 100 person-years. The use of healthcare services and the length of stays in hospital wards were also measured in affected patients. Given its multi-system involvement and progressively deteriorating course, all patients with SMA1 and SMA2, and some patients with SMA3 at the later stage of disease required frequent admissions and consultations by multiple subspecialties and allied health professionals. Our study confirmed the strikingly high healthcare burden and economic costs of SMA1 especially, followed by SMA2 and SMA3.

The results of our analysis show that the healthcare costs of SMA-related direct inpatient and outpatient costs are extremely high. As expected, the costs are highest for patients with SMA1, the most severe form of the disease. The 18-year cumulative inpatient costs in Hong Kong for identified cases of SMA1 exceed HK$ 18,500,000 (US$2,371,795) per patient. By comparison, the 18-year cumulative costs for SMA2 and SMA3 per patient were 17.5% and 1.7% of that for SMA1. Our result of high economic burden of SMA was consistent with those reported in overseas studies [21–24]. The estimated annual cost of SMA in Australia is US$143,705 per household, ranging from US$94,948 for SMA3 to US$229,346 for SMA1[22]. Similarly, a cohort study in Taiwan showed that the annualized mean inpatient and outpatient cost for all types of SMA patients was as high as US$47,862 [25]. Data from US also suggested the substantial medical costs incurred by patients with SMA [23, 24]. The total mean healthcare costs for patient with SMA1 and other SMA were US$137,627 per-patient-per-year and US76,371 per-patient-per-year, respectively [24]. When comparing with patients without SMA, patients with SMA usually had > 50-fold higher inpatient and outpatient costs [23]. While the enormous economic burden of SMA is acknowledged, direct comparisons of the specific costs across studies are not feasible. It is not only because the study design and models are different from one study to another, but also the results from each study are largely affected by geographical factors, for example, different healthcare system and costing, and standard of care practice.

In Hong Kong, all the pediatric patients with SMA are taken care by a multidisciplinary team in their hospitals. The team included pediatric specialists of neurology, respiratory, orthopaedic surgery, and allied health professionals. For those with feeding problems, the support of pediatric surgery, a centralised service, would also be provided. Most patients have regular followed-ups every 4–6 months. The follow-ups were arranged either as multidisciplinary joint clinics or as day admissions in the general ward for the team evaluation. All patients with SMA1 and most patients with SMA2 studied in special schools for children with physical impairment. At schools, all patients were attended by school physiotherapists and occupational therapists for the daily exercise program appropriate to their motor functional status. For those with feeding problems, they would also be attended by the school speech therapist for their oromotor training. This could explain why our patients have a low attendance of isolated allied health clinic in the hospital. The low attendance of A&E departments could be explained by the fact that some patients could be directly admitted to the general ward when they got sick after the families had informed the paediatric team.

Before the availability of SMA novel therapies, all families with children with SMA1 would be counselled about the grave prognosis and the introducing of redirection of care with end-of-life comfort care. The management decisions were family centered. Some families opted for palliative care, while others preferred intensive management including ventilation support, gastrostomy, spinal fusion, and comprehensive therapy training for their child. The SMA mortality rate in our study was highest in the SMA1 group with a mean age of death at 1.6 years and median age of death at 0.8 years. Overseas studies also showed early dying for SMA1 with median age of death ranged from 0.6 to 0.7 years [10, 11, 25] to 1.1 years [26]. However, it should be noted that the range of survival times for patients with SMA1 may be wide. This can be explained by the different management approaches from letting the nature takes its course to early starting of ventilation support, as well as the patients had variable combination of SMA type 1 subtypes [27] and *SMN2* copy numbers [28]. With the advancement in medical technology and care, the survival time of patients with SMA has been increasing over the years [27–29].

To date, three novel therapies for SMA have been approved by the US FDA. However, these drugs are among the most expensive in medicine. Onasemnogene abeparvovec-xioi (Zolgensma®) is a novel gene therapy for pediatric patients under 2 years of age with SMA with bi-allelic mutations in *SMN1* and has an expected unit price of US$2.15 million [30]. Nusinersen (Spinraza®) is a survival motor neuron-2 gene-directed antisense oligonucleotide for both pediatric and adult SMA patients, and it is priced at US$750.000 for the first year of therapy and US$375,000 for treatments in subsequent years [31]. Risdiplam (Evrysdi™), the third drug approved by FDA for the treatment of SMA [32], has expected costs as high as US$340,000 per year [33]. Due to the overwhelming costs, the novel therapies listed above are unlikely to be cost-effective in most parts of the world [34, 35]. Moreover, in any cost-effectiveness study, it is also difficult or impossible to give an estimated cost of a human life.

In Hong Kong, all the pediatric patients with SMA1 received intrathecal nusinersen treatment since 2018 under the Extended Access Program [29]. Since 2019, all the pediatric patients with SMA1, SMA2 and SMA3 that fulfilled the treatment criteria received nusinersen supported by a government endorsed Ultra-Expansive Drug Subsidy Program for treating uncommon disorders under the Community Care Fund. The patients must be a Hong Kong citizen and are followed up in hospitals under the Hong Kong Hospital Authority. Thirty-one applications had been approved by Feb 2021 for paediatric patients, and US$10.87 million of subsidy have been granted. Each family must also pass a ‘household-based’ financial assessment conducted by medical social workers to decide on their financial contribution. The maximum contribution amount for a family is US$ 0.13 million (HK$ 1 million) each year. Recently, the oral risdiplam compassionate use program has also been started for some of our SMA1 and SMA2 patients with intrathecal access problem. All the patients started treatment at the symptomatic stage. Currently newborn screening is not available in Hong Kong.

There is cumulative strong evidence that the earlier the treatment, the better the clinical outcome. A study investigating infants with mostly SMA1 or SMA2 having either 2 or 3 *SMN2* copies, as identified by newborn screening, showed that of those who started nusinersen in the pre-symptomatic stage, 100% achieved independent sitting, 92% achieved walking with assistance, and 88% achieved independent walking [36]. In separate studies, following onasemnogene abeparvovec-xioi [17], risdiplam [18] and nusinersen [28] treatment, better clinical outcomes, such as higher motor gain and event-free survival, were seen in SMA1 infants who were treated at an earlier symptomatic stage before 3 months of age.

SMA newborn screening (NBS) allows the identification of affected newborn babies early in life and the initiation of novel SMA disease-modifying treatments at the pre-symptomatic or early symptomatic stage. Currently, NBS for SMA is performed in nine countries and is expected to expand globally rapidly in the coming years. However, the lack of abundant cost/benefit data has been identified as one of the major obstacles to SMA NBS advocacy and implementation [37]. Health economic evaluations on SMA NBS started emerging in recent years [38]. An economic evaluation from Australia have confirmed the cost-effectiveness of NBS combined with gene therapy when comparing with no screening or late nusinersen treatment by gaining 85 quality-adjusted life years (QALYs) and saving US$2.4 million per 100,000 babies screened [38]. In addition, a costing-analysis found that the direct medical costs of patients with early treatment (treatment provided before symptoms presented) were significantly lower than those with late treatment. This study supported that early identification and treatment for patients with SMA can reduce the costs (39). Our analysis provides accurate real-world evidence of the extremely high healthcare costs from the significant healthcare service utilization among pediatric SMA patients before the availability of these disease-modifying treatments.

## Strength and limitation

This study generates knowledge on the socioeconomic burden, healthcare cost and mortality from SMA—a medically complex disease with a degenerative course, in our paediatric age. This study uses real-world data of the SMA patients to provide mortality data and the actual economic burden of pediatric patients with SMA. All patients included in this study were under the care of HA, which provides healthcare services in all public hospitals, general and special out-patient clinics in Hong Kong. As the ongoing healthcare costs of SMA are understandably high, over 99% of our SMA patients attended HA public hospitals and the multi-specialties clinics there. This means that our study findings provide highly reliable territory wide real-world data. However, several limitations should be acknowledged. First, the current findings are likely to underestimate the healthcare costs, as for those multidisciplinary clinics or day admissions, many of the individual specialty consultation sessions could not be counted. Moreover, the possible underestimation of the costs may also be caused by the absence of the assessment of the costs incurred by patients who were censored due to treatment switch. These patients may incur more costs if they did not transfer the treatment. Second, we have not addressed the other direct costs associated with SMA, including the rehabilitation equipment and special education needs, and other indirect costs including loss of quality of life of the SMA patients, the caregiver burden, and lost productivity. Lastly, less than 10 patients were able to survive to 13 years, possibly resulting in the results on costs and healthcare resource utilization at later years biased.

## Conclusion

Using long follow-up real-world data, this study confirmed that the mortality rates and healthcare expenditures of direct inpatient and outpatient costs related to SMA without novel treatment were extremely high. The available disease-modifying treatments need to be given in a timely fashion to alter the disease course and prevent the development of severe disease and disability. This is beneficial not only to patients but also to the healthcare systems.

The advent of novel disease-modifying treatments for pediatric patients with SMA is rapidly remodeling clinical care and advancing patient outcomes. The strong scientific evidence from clinical trials and real-world studies have shown impactful and meaningful improvements in survival, motor function, and motor milestone gains in infants and children. The best motor outcomes are seen in affected babies who receive treatment in the pre-symptomatic stage. Available evidence shows that the best clinical outcomes are in patients with SMA who are treated at the pre- or early symptomatic stage. These findings provide compelling and cost-effective evidence that support NBS for SMA. Newborn screenings enable identification of affected babies in the asymptomatic stage. Through NBS and early disease-modifying treatments, the extremely high SMA-associated healthcare costs can be effectively minimized, and the significant disability and high mortality of the disease can also be prevented.

### Supplementary Information

Below is the link to the electronic supplementary material.

Supplementary file1 (DOCX 57 KB)

## Abbreviations

A&E: Accident and emergency
DMTs: Disease-modifying treatments
FDA: Food and drug administration
HA: Hospital authority
HDU: High-dependency units
HKD: Hong Kong dollars
HRs: Hazard ratios
ICU: Intensive care units
IRs: Incidence rates
NBS: Newborn screening
SMA: Spinal muscular atrophy
SMA1: SMA type 1
SMA2: SMA type 2
SMA3: SMA type 3
SMN1: Survival motor neuron 1
SMN2: Survival motor neuron 2
SOPC: Special outpatient clinics
USD: United States dollar
