# Supplementary material for: Significant healthcare burden and life cost of spinal muscular atrophy: real-world data
Source: Eur J Health Econ. 2022 Nov 20;24(8):1373–82. doi: 10.1007/s10198-022-01548-5 (PMC10533630; doi:10.1007/s10198-022-01548-5)
Supplement: Supplementary file 1 — Supplementary file1 (DOCX 57 KB) [file 10198_2022_1548_MOESM1_ESM.docx]

Supplementary Table 1 Unit costs of Healthcare Services

| **Items** | **Cost (HK$)** |
| --- | --- |
| **Healthcare services**^‡^ |  |
| Specialist outpatient clinic | 1,190 per visit |
| Accident and emergency | 1,230 per visit |
| Allied health professionals |  |
| Speech therapy | 550 per visit |
| Physiotherapy | 550 per visit |
| Occupational therapy | 550 per visit |
| Medical social worker | 550 per visit |
| Hospitalization |  |
| General ward | 5,100 per day |
| Intensive care unit | 24,400 per day |
| High dependency unit | 13,650 per day |
| **Procedures**^§^ |  |
| Posterior dorsal fusion | 29,688 |
| Posterior lumbar fusion | 80,175 |
| Spinal fusion | 116,020 |
| Spinal tap | 9,410 |
| Spinal canal injection | 9,410 |
| Gastrostomy | 34,125 |
| Temporary / Permanent tracheostomy | 16,050 |
| Indwelling catheterisation | 16,050 |
| Femoral wedge osteotomy | 43,950 |
| Laparotomy | 45,775 |
| Venous catheter | 9,410 |
| Arterial catheterisation | 9,410 |
| Cholecystostomy | 27,940 |
| Achillotenotomy | 24,900 |
| Episiotomy | 28,953 |
| Tenotomy | 39,188 |
| Glossotomy | 16,050 |
| Sphincterotomy | 31,783 |
| Dialysis | 20,288 |
| Spinal canal catheter insertation | 24,900 |
| Appendicectomy | 34,125 |
| Tonsillectomy | 29,513 |
| Notes:  ‡ Data source: 2017 Hong Kong SAR Government Gazette and Hospital Authority Ordinance (chapter 113): Public charges – non-eligible persons. Available from: https://www3.ha.org.hk/fnc/Operations.aspx?lang=ENG | |
| § Data source: 2017 Hong Kong SAR Government Gazette and Hospital Authority Ordinance (chapter 113): Private charges (midpoint value) Charge for operations | |

Supplementary Table 2. Costs of healthcare services per patient with SMA by age.

|  |  | SMA Type | | |  | Special outpatient clinic | | Accident & Emergency | | Allied health | | Healthcare services | | Operation or procedures | |
| --- | --- | --- | --- | --- | --- | --- | --- | --- | --- | --- | --- | --- | --- | --- | --- |
| Age | N | 1 | 2 | 3 | Person-days | Mean | 95% CI | Mean | 95% CI | Mean | 95% CI | Mean | 95% CI | Mean | 95% CI |
| 0 | 71 | 34 | 27 | 10 | 23453 | 7559 | (4,970, 10,148) | 1646 | (1,006, 2,285) | 256 | (90, 422) | 9460 | (6,321, 12,599) | 5202 | (1,447, 8,957) |
| 1 | 55 | 18 | 27 | 10 | 19490 | 17223 | (11,469, 22,976) | 917 | (487, 1,347) | 230 | (106, 354) | 18369 | (12,443, 24,296) | 4434 | (1,380, 7,488) |
| 2 | 50 | 14 | 27 | 9 | 17956 | 23752 | (16,020, 31,484) | 1205 | (679, 1,731) | 352 | (58, 646) | 25310 | (17,423, 33,196) | 5442 | (-567, 11,452) |
| 3 | 47 | 11 | 27 | 9 | 16414 | 20103 | (12,329, 27,878) | 1387 | (754, 2,020) | 117 | (11, 223) | 21607 | (13,756, 29,459) | 4455 | (1,517, 7,393) |
| 4 | 44 | 9 | 26 | 9 | 15199 | 14956 | (8,942, 20,970) | 1733 | (778, 2,689) | 250 | (99, 401) | 16939 | (10,764, 23,115) | 9019 | (3,296, 14,741) |
| 5 | 38 | 7 | 22 | 9 | 13259 | 21076 | (13,113, 29,038) | 1489 | (632, 2,346) | 145 | (14, 276) | 22709 | (14,614, 30,804) | 8340 | (1,325, 15,355) |
| 6 | 33 | 6 | 18 | 9 | 11629 | 15182 | (10,483, 19,880) | 1901 | (799, 3,003) | 317 | (-133, 766) | 17399 | (12,405, 22,393) | 4193 | (-172, 8,558) |
| 7 | 30 | 5 | 17 | 8 | 10418 | 12614 | (7,847, 17,381) | 1763 | (477, 3,049) | 367 | (-56, 789) | 14744 | (9,496, 19,991) | 9445 | (-1,486, 20,376) |
| 8 | 27 | 5 | 16 | 6 | 8822 | 13310 | (8,117, 18,503) | 1731 | (507, 2,955) | 224 | (-54, 502) | 15266 | (9,481, 21,050) | 8187 | (-315, 16,689) |
| 9 | 23 | 4 | 14 | 5 | 7975 | 10400 | (6,431, 14,368) | 1658 | (263, 3,052) | 167 | (-95, 430) | 12225 | (7,763, 16,687) | 5422 | (-1,240, 12,084) |
| 10 | 21 | 4 | 12 | 5 | 7094 | 11957 | (5,076, 18,837) | 1581 | (99, 3,064) | 183 | (-59, 425) | 13721 | (6,423, 21,020) | 15722 | (-1,597, 33,041) |
| 11 | 17 | 3 | 9 | 5 | 5847 | 11200 | (4,617, 17,783) | 796 | (91, 1,501) | 291 | (-134, 717) | 12287 | (4,935, 19,639) | 0 | NA |
| 12 | 15 | 2 | 8 | 5 | 4968 | 9599 | (5,657, 13,541) | 328 | (16, 640) | 73 | (-34, 181) | 10001 | (5,872, 14,130) | 3579 | (-1,915, 9,073) |
| 13 | 9 | 1 | 5 | 3 | 3071 | 7140 | (2,321, 11,959) | 683 | (-385, 1,752) | 122 | (-64, 309) | 7946 | (2,606, 13,285) | 2829 | (-3,695, 9,352) |
| 14 | 8 | 1 | 4 | 3 | 2920 | 10710 | (3,615, 17,805) | 154 | (-210, 517) | 0 | NA | 10864 | (3,537, 18,191) | 0 | NA |
| 15 | 8 | 1 | 4 | 3 | 2765 | 8330 | (318, 16,342) | 0 | NA | 0 | NA | 8330 | (318, 16,342) | 18852 | (-18,040, 55,744) |
| 16 | 7 | 1 | 3 | 3 | 2384 | 12750 | (1,897, 23,603) | 0 | NA | 0 | NA | 12750 | (1,897, 23,603) | 0 | NA |
| 17 | 5 | 1 | 3 | 1 | 1551 | 22848 | (-23,788, 69,484) | 984 | (-1,748, 3,716) | 330 | (-586, 1,246) | 24162 | (-26,083, 74,407) | 24239 | (-30,932, 79,410) |
| 18 | 1 | 1 | 0 | 0 | 365 | 34510 | NA | 6150 | NA | 2750 | NA | 43410 | NA | 34125 | NA |
| Total |  |  |  |  |  | 285,218 |  | 26,106 |  | 6,174 |  | 317,499 |  | 163,485 |  |

| Age |  | General wards | | Intensive Care Units | | High-dependency Units | | Hospitalization | | Total | | Cumulative Total |
| --- | --- | --- | --- | --- | --- | --- | --- | --- | --- | --- | --- | --- |
|  | N | Mean | 95% CI | Mean | 95% CI | Mean | 95% CI | Mean | 95% CI | Mean | 95% CI | Mean |
| 0 | 71 | 111338 | (67,668, 155,009) | 562918 | (258,045, 867,791) | 10766 | (-597, 22,129) | 685023 | (366,777, 1,003,269) | 699685 | (379,249, 1,020,120) | 699685 |
| 1 | 55 | 27633 | (6,862, 48,403) | 698727 | (168,797, 1,228,658) | 9927 | (-2,666, 22,520) | 736287 | (201,723, 1,270,851) | 759091 | (222,915, 1,295,267) | 1458776 |
| 2 | 50 | 60486 | (15,573, 105,399) | 467992 | (31,718, 904,266) | 273 | (-276, 822) | 528751 | (82,753, 974,749) | 559503 | (112,469, 1,006,537) | 2018279 |
| 3 | 47 | 52845 | (2,194, 103,495) | 417915 | (4,074, 831,756) | 290 | (-294, 875) | 471050 | (39,299, 902,801) | 497113 | (66,010, 928,216) | 2515392 |
| 4 | 44 | 75051 | (10,251, 139,851) | 311932 | (61,950, 561,914) | 3102 | (-2,029, 8,233) | 390085 | (98,276, 681,895) | 416043 | (124,351, 707,735) | 2931435 |
| 5 | 38 | 71668 | (7,760, 135,577) | 168232 | (-70,534, 406,997) | 8980 | (-4,018, 21,979) | 248880 | (-40,853, 538,614) | 279929 | (-10,312, 570,171) | 3211364 |
| 6 | 33 | 92032 | (-7,648, 191,712) | 449921 | (-156,199, 1,056,042) | 9927 | (-10,294, 30,148) | 551880 | (-83,445, 1,187,205) | 573473 | (-61,918, 1,208,864) | 3784837 |
| 7 | 30 | 98430 | (-12,507, 209,367) | 319640 | (-52,556, 691,836) | 25025 | (-22,452, 72,502) | 443095 | (4,052, 882,138) | 467283 | (25,119, 909,448) | 4252120 |
| 8 | 27 | 122211 | (-27,158, 271,581) | 46089 | (-9,959, 102,137) | 30839 | (-25,468, 87,146) | 199139 | (-4,204, 402,482) | 222591 | (14,537, 430,646) | 4474712 |
| 9 | 23 | 121070 | (-50,053, 292,192) | 43496 | (6,675, 80,316) | 46885 | (-50,348, 144,118) | 211450 | (-17,803, 440,703) | 229097 | (-5,268, 463,462) | 4703808 |
| 10 | 21 | 138914 | (-46,326, 324,154) | 148724 | (-28,285, 325,733) | 35750 | (-34,587, 106,087) | 323388 | (65,818, 580,959) | 352831 | (94,329, 611,334) | 5056640 |
| 11 | 17 | 180900 | (-71,846, 433,646) | 4306 | (-2,326, 10,937) | 40147 | (-44,961, 125,255) | 225353 | (-72,388, 523,094) | 237640 | (-63,407, 538,687) | 5294280 |
| 12 | 15 | 127160 | (-132,315, 386,635) | 19520 | (-8,546, 47,586) | 0 | NA | 146680 | (-139,866, 433,226) | 160260 | (-125,228, 445,748) | 5454540 |
| 13 | 9 | 219867 | (-255,237, 694,970) | 157244 | (-82,780, 397,269) | 0 | NA | 377111 | (-121,842, 876,064) | 387886 | (-113,695, 889,466) | 5842425 |
| 14 | 8 | 238425 | (-309,982, 786,832) | 0 | NA | 0 | NA | 238425 | (-309,982, 786,832) | 249289 | (-300,831, 799,408) | 6091714 |
| 15 | 8 | 228225 | (-296,009, 752,459) | 39650 | (-46,162, 125,462) | 10238 | (-9,762, 30,237) | 278113 | (-351,403, 907,628) | 305294 | (-366,611, 977,199) | 6397008 |
| 16 | 7 | 268114 | (-377,598, 913,827) | 0 | NA | 0 | NA | 268114 | (-377,598, 913,827) | 280864 | (-368,098, 929,826) | 6677873 |
| 17 | 5 | 61200 | (-94,752, 217,152) | 9760 | (-17,338, 36,858) | 0 | NA | 70960 | (-112,061, 253,981) | 119361 | (-167,944, 406,666) | 6797234 |
| 18 | 1 | 257550 | NA | 280600 | NA | 0 | NA | 538150 | NA | 615685 | NA | 7412919 |
| Total |  | 2,553,119 |  | 4,146,666 |  | 232,150 |  | 6,931,934 |  | 7,412,919 |  |  |

Notes: SMA = spinal muscular atrophy; NA = Not available; CI = confidence interval. The cost is reported in HKD.

Supplementary Table 3. Utilization frequency of healthcare services per patient with SMA by age

|  |  | SMA Type | | |  | Specialist Outpatient Clinic | | Accident & Emergency | | Allied health | | Healthcare services | |
| --- | --- | --- | --- | --- | --- | --- | --- | --- | --- | --- | --- | --- | --- |
| Age | N | 1 | 2 | 3 | Person-days | Mean | 95% CI | Mean | 95% CI | Mean | 95% CI | Mean | 95% CI |
| 0 | 71 | 34 | 27 | 10 | 23453 | 6 | (4, 9) | 1 | (1, 2) | 0 | (0, 1) | 8 | (5, 11) |
| 1 | 55 | 18 | 27 | 10 | 19490 | 14 | (10, 19) | 1 | (0, 1) | 0 | (0, 1) | 16 | (11, 21) |
| 2 | 50 | 14 | 27 | 9 | 17956 | 20 | (13, 26) | 1 | (1, 1) | 1 | (0, 1) | 22 | (15, 28) |
| 3 | 47 | 11 | 27 | 9 | 16414 | 17 | (10, 23) | 1 | (1, 2) | 0 | (0, 0) | 18 | (12, 25) |
| 4 | 44 | 9 | 26 | 9 | 15199 | 13 | (8, 18) | 1 | (1, 2) | 0 | (0, 1) | 14 | (9, 20) |
| 5 | 38 | 7 | 22 | 9 | 13259 | 18 | (11, 24) | 1 | (1, 2) | 0 | (0, 1) | 19 | (12, 26) |
| 6 | 33 | 6 | 18 | 9 | 11629 | 13 | (9, 17) | 2 | (1, 2) | 1 | (0, 1) | 15 | (11, 19) |
| 7 | 30 | 5 | 17 | 8 | 10418 | 11 | (7, 15) | 1 | (0, 2) | 1 | (0, 1) | 13 | (8, 17) |
| 8 | 27 | 5 | 16 | 6 | 8822 | 11 | (7, 16) | 1 | (0, 2) | 0 | (0, 1) | 13 | (8, 18) |
| 9 | 23 | 4 | 14 | 5 | 7975 | 9 | (5, 12) | 1 | (0, 2) | 0 | (0, 1) | 10 | (7, 14) |
| 10 | 21 | 4 | 12 | 5 | 7094 | 10 | (4, 16) | 1 | (0, 2) | 0 | (0, 1) | 12 | (5, 18) |
| 11 | 17 | 3 | 9 | 5 | 5847 | 9 | (4, 15) | 1 | (0, 1) | 1 | (0, 1) | 11 | (4, 17) |
| 12 | 15 | 2 | 8 | 5 | 4968 | 8 | (5, 11) | 0 | (0, 1) | 0 | (0, 0) | 8 | (5, 12) |
| 13 | 9 | 1 | 5 | 3 | 3071 | 6 | (2, 10) | 1 | (0, 1) | 0 | (0, 1) | 7 | (2, 11) |
| 14 | 8 | 1 | 4 | 3 | 2920 | 9 | (3, 15) | 0 | (0, 0) | 0 | NA | 9 | (3, 15) |
| 15 | 8 | 1 | 4 | 3 | 2765 | 7 | (0, 14) | 0 | NA | 0 | NA | 7 | (0, 14) |
| 16 | 7 | 1 | 3 | 3 | 2384 | 11 | (2, 20) | 0 | NA | 0 | NA | 11 | (2, 20) |
| 17 | 5 | 1 | 3 | 1 | 1551 | 19 | (-20, 58) | 1 | (-1, 3) | 1 | (-1, 2) | 21 | (-22, 64) |
| 18 | 1 | 1 | 0 | 0 | 365 | 29 | NA | 5 | NA | 5 | NA | 39 | NA |

|  |  | SMA Type | | |  | Operation or procedures | | General wards | | Intensive Care Units | | High-dependency Units | | Hospitalization | |
| --- | --- | --- | --- | --- | --- | --- | --- | --- | --- | --- | --- | --- | --- | --- | --- |
| Age | N | 1 | 2 | 3 | Person-days | Mean | 95% CI | Mean | 95% CI | Mean | 95% CI | Mean | 95% CI | Mean | 95% CI |
| 0 | 71 | 34 | 27 | 10 | 23453 | 0 | (0, 1) | 22 | (13, 30) | 23 | (11, 36) | 1 | (0, 2) | 46 | (29, 62) |
| 1 | 55 | 18 | 27 | 10 | 19490 | 0 | (0, 0) | 5 | (1, 9) | 29 | (7, 50) | 1 | (0, 2) | 35 | (12, 58) |
| 2 | 50 | 14 | 27 | 9 | 17956 | 0 | (0, 1) | 12 | (3, 21) | 19 | (1, 37) | 0 | (0, 0) | 31 | (10, 52) |
| 3 | 47 | 11 | 27 | 9 | 16414 | 0 | (0, 1) | 10 | (0, 20) | 17 | (0, 34) | 0 | (0, 0) | 28 | (5, 50) |
| 4 | 44 | 9 | 26 | 9 | 15199 | 1 | (0, 1) | 15 | (2, 27) | 13 | (3, 23) | 0 | (0, 1) | 28 | (7, 48) |
| 5 | 38 | 7 | 22 | 9 | 13259 | 0 | (0, 1) | 14 | (2, 27) | 7 | (-3, 17) | 1 | (0, 2) | 22 | (1, 42) |
| 6 | 33 | 6 | 18 | 9 | 11629 | 0 | (0, 1) | 18 | (-1, 38) | 18 | (-6, 43) | 1 | (-1, 2) | 37 | (2, 72) |
| 7 | 30 | 5 | 17 | 8 | 10418 | 0 | (0, 1) | 19 | (-2, 41) | 13 | (-2, 28) | 2 | (-2, 5) | 34 | (1, 67) |
| 8 | 27 | 5 | 16 | 6 | 8822 | 1 | (0, 1) | 24 | (-5, 53) | 2 | (0, 4) | 2 | (-2, 6) | 28 | (-3, 60) |
| 9 | 23 | 4 | 14 | 5 | 7975 | 0 | (0, 1) | 24 | (-10, 57) | 2 | (0, 3) | 3 | (-4, 11) | 29 | (-7, 65) |
| 10 | 21 | 4 | 12 | 5 | 7094 | 1 | (0, 1) | 27 | (-9, 64) | 6 | (-1, 13) | 3 | (-3, 8) | 36 | (-1, 73) |
| 11 | 17 | 3 | 9 | 5 | 5847 | 0 | NA | 35 | (-14, 85) | 0 | (0, 0) | 3 | (-3, 9) | 39 | (-14, 91) |
| 12 | 15 | 2 | 8 | 5 | 4968 | 0 | (0, 1) | 25 | (-26, 76) | 1 | (0, 2) | 0 | NA | 26 | (-26, 78) |
| 13 | 9 | 1 | 5 | 3 | 3071 | 0 | (0, 1) | 43 | (-50, 136) | 6 | (-3, 16) | 0 | NA | 50 | (-43, 142) |
| 14 | 8 | 1 | 4 | 3 | 2920 | 0 | NA | 47 | (-61, 154) | 0 | NA | 0 | NA | 47 | (-61, 154) |
| 15 | 8 | 1 | 4 | 3 | 2765 | 1 | (0, 1) | 45 | (-58, 148) | 2 | (-2, 5) | 1 | (-1, 2) | 47 | (-61, 155) |
| 16 | 7 | 1 | 3 | 3 | 2384 | 0 | NA | 53 | (-74, 179) | 0 | NA | 0 | NA | 53 | (-74, 179) |
| 17 | 5 | 1 | 3 | 1 | 1551 | 1 | (-1, 3) | 12 | (-19, 43) | 0 | (-1, 2) | 0 | NA | 12 | (-19, 44) |
| 18 | 1 | 1 | 0 | 0 | 365 | 1 | NA | 51 | NA | 12 | NA | 0 | NA | 62 | NA |

Notes: SMA = spinal muscular atrophy; NA = Not available; CI = confidence interval
